# Obesity and Insulin Resistance Are the Main Determinants of Postprandial Lipoprotein Dysmetabolism in Polycystic Ovary Syndrome

**DOI:** 10.1155/2016/9545239

**Published:** 2016-02-18

**Authors:** Tommy Kyaw Tun, Anne McGowan, Niamh Phelan, Neuman Correia, Gerard Boran, Anna-Louise O'Connor, Helen M. Roche, James Gibney

**Affiliations:** ^1^Department of Endocrinology and Diabetes, Tallaght Hospital, Tallaght, Dublin 24, Ireland; ^2^Department of Chemical Pathology, Tallaght Hospital, Tallaght, Dublin 24, Ireland; ^3^Nutrigenomics Research Group, UCD Conway Institute of Biomolecular and Biomedical Research, School of Public Health and Population Science, University College Dublin, Belfield, Dublin 4, Ireland

## Abstract

Postprandial dyslipidaemia may be a plausible mechanism by which polycystic ovary syndrome (PCOS) increases cardiovascular risk. We sought to investigate whether the postprandial glucose and insulin and lipid and lipoprotein responses, including that of apolipoprotein B-48 (apoB-48) containing chylomicrons, to a mixed meal are different in obese PCOS women when compared to obese control subjects and whether differences, if any, are related to obesity, insulin resistance (IR), hyperandrogenaemia, or PCOS status. 26 women with PCOS (age 30.4 ± 1.2 years (mean ± SEM), body mass index (BMI) 36.8 ± 1.5 kg/m^2^) and 26 non-PCOS subjects (age 34.1 ± 0.9 years, BMI 31.5 ± 1.0 kg/m^2^) were studied before and up to 8 hours following a standard mixed meal. AUC-triglyceride (AUC-TG) was higher and AUC-high-density lipoprotein (AUC-HDL) lower in PCOS women. These differences were not apparent when BMI was accounted for. Insulin sensitivity (*S*_I_), AUC-apoB-48, and AUC-apolipoprotein B (AUC-apoB) were found to be independent predictors of AUC-TG, accounting for 55% of the variance. Only AUC-insulin remained significantly elevated following adjustment for BMI. Obesity related IR explains postprandial hypertriglyceridaemia and hyperinsulinaemic responses. Management of obesity in premenopausal women with PCOS is likely to reduce their cardiovascular risk burden.

## 1. Introduction

There is considerable evidence from epidemiological research, meta-analysis [1, 2], and prospective clinical trials [3, 4] to support an independent role for fasting and postprandial plasma triglycerides (TG) as a risk factor for cardiovascular disease (CVD). Increased plasma remnants of triglyceride-rich lipoproteins (TRLs), caused by delayed elimination of the same, has been shown to be prospectively associated with angiographic evidence of atherosclerosis and cardiac events [5]. Postprandial hypertriglyceridaemia is associated with two other well established cardiovascular risks—IR and obesity—both of which are prevalent in PCOS. An increase in postprandial lipoproteins is often found in IR states [6] and hyperinsulinaemia itself appears to delay and exacerbate postprandial accumulation of intestinally derived chylomicrons [7]. Obesity, especially visceral adiposity, also contributes to a magnified postprandial TG response [8, 9].

Postprandial hypertriglyceridaemia may be a plausible mechanism by which PCOS increases cardiovascular risk. Whilst numerous studies have investigated plasma lipid profile under fasting conditions in women with PCOS, few have investigated changes in the postprandial setting. In a previous study by Velázquez M et al. a strong positive correlation between postprandial TG and increasing waist-to-hip ratio (WHR) was demonstrated [10]. That study evaluated overweight (BMI 27.41 ± 0.50 kg/m^2^) PCOS women and compared them to lean PCOS women and lean control subjects. There were no obese controls and there was no analysis of the influence of androgens on postprandial hypertriglyceridaemia [10]. A separate study by Bahceci et al. evaluated postprandial responses to an oral fat tolerance test, comparing lean PCOS women with lean controls (BMI 23.5 ± 2.6 kg/m^2^ versus 23.1 ± 4.0 kg/m^2^; *p* > 0.05) [11]. There were no obese or overweight controls in this study. They showed that PCOS women had higher baseline insulin levels, IR as assessed by homeostatic model assessment-IR (HOMA_IR_), AUC-TG, AUC-total-cholesterol, AUC-very-low-density lipoprotein cholesterol (AUC-VLDL-chol), and AUC-apoB. Surprisingly, though, AUC-insulin did not differ between the two groups. As with the study by Velázquez M et al., no analyses were reported between androgens and postprandial lipids [11].

It is also unclear whether postprandial hypertriglyceridaemia is mainly due to the contribution of intestinally derived apoB-48 containing chylomicrons or hepatically derived apoB-100 containing VLDL particles. Both VLDL and chylomicrons share a common lipolytic pathway and are hydrolysed by lipoprotein lipase (LPL), an enzyme predominantly found on the endothelial surfaces of the capillaries of adipose tissue, heart, and skeletal muscle. Hydrolysis results in the formation of a spectrum of smaller, denser particles. There are a number of described receptor mediated pathways through which these particles may eventually be cleared from the circulation [12]. Traditionally it has been difficult to assess what proportions of TRLs are chylomicrons and VLDL. However, using ELISA, it is now possible to directly measure the quantity of apoB-48 and hence chylomicron particles, in whole plasma [13, 14].

We sought to investigate whether the postprandial glucose and insulin and lipid and lipoprotein responses, including that of apolipoprotein B-48 (apoB-48) containing chylomicrons, to a mixed meal are different in obese PCOS women when compared to obese control subjects and whether differences, if any, are related to obesity, insulin resistance (IR), hyperandrogenaemia, or PCOS status.

## 2. Methods

### 2.1. Subjects

Fifty-two obese premenopausal women with (*n* = 26) and without (*n* = 26) PCOS were recruited. Women with PCOS were recruited from the endocrinology outpatient clinics (Tallaght Hospital, Dublin, Ireland). Normal women were recruited by local advertisement. PCOS was defined according to the National Institute of Health (NIH) criteria as chronic oligomenorrhea (fewer than nine menstrual cycles per year) and clinical and/or biochemical evidence of hyperandrogenism, in the absence of other disorders causing the same phenotype [15]. Clinical criteria included hirsutism with a Ferriman-Gallwey score greater than 9, acne, or male pattern alopecia; biochemical criteria included total testosterone, androstenedione, or dehydroepiandrosterone sulphate (DHEAS) greater than the laboratory reference range. All normal subjects were eumenorrheic with testosterone levels within the normal range and were studied in the follicular phase of the menstrual cycle. Subjects were excluded if they were younger than 18 years old or older than 45 years old; were non-Caucasian, pregnant, or lactating; had a recent or chronic illness or medication likely to influence results; or were taking any medications likely to influence the results including hormonal contraception, antihypertensives, lipid-lowering medications, and antiplatelet or anti-inflammatory agents. Fourteen women in the PCOS group and 3 women without PCOS had a first-degree relative with type 2 diabetes mellitus. All study subjects gave their written signed consent to the study, which was approved by the Research Ethics Committee of Tallaght Hospital and St. James's Hospital (Dublin, Ireland).

### 2.2. Clinical Protocol

This was a cross-sectional study. All subjects had their height measured with a Harpenden stadiometer and weight measured in light clothing. BMI was calculated as weight (kg)/height squared (m^2^). Waist circumference (WC) and hip circumference (HC) were measured with a nondistensible flexible tape, and WHR was calculated accordingly. All subjects also underwent estimation of body composition including percentage body fat (%BF) and percentage lean mass (%LM) using bioimpedance analysis using the Bodystat 1500 system (Bodystat Ltd., UK).

### 2.3. Frequently Sampled Intravenous Glucose Tolerance Test (fsIVGTT)

Subjects attended the Clinical Investigation Unit after a 12-hour overnight fast and were requested to refrain from vigorous exercise and alcohol on the day prior to their fsIVGTT. On the morning of the fsIVGTT, two cannulae were inserted into the antecubital veins of both forearms. A fasting blood sample was taken from cannula 1. Then a bolus of 50% glucose solution (0.3 g/kg body weight (BW)) was infused into cannula 2 over a 1-minute period followed by 20 mL of 0.9% saline. Twenty minutes later, a dose of insulin (0.03 U/kg BW) was infused into the same cannula (cannula 2), which was then removed at 30 min after initiation of glucose administration. Meanwhile, blood was sampled through cannula 1 at frequent intervals over a 3-hour period (−5, 0, 2, 4, 8, 19, 22, 30, 40, 50, 70, 90, and 180 min after the start of the glucose injection) for determination of glucose and insulin at each time point. Blood samples were spun at 3000 rpm for 10 min after which the plasma was aliquoted and stored at −80°C until required for future analysis. Insulin sensitivity (*S*_I_), glucose effectiveness (*S*_G_), acute insulin response (AIR_G_), and disposition index (DI) were estimated using the MINMOD computer program (version 3.0, copyright R. N. Bergman).

### 2.4. Mixed Meal and Sampling

Following a 12-hour overnight fast, subjects came to the Clinical Investigation Unit in Tallaght Hospital for blood sampling and a high calorific meal. The mixed meal was designed and analyzed by a qualified dietician. It consisted of 948 kcal. 48% of the total calories were derived from fat (20% saturated, 16% monounsaturated, and 8% polyunsaturated). 36%  and  15% of the calories were derived from carbohydrate and protein, respectively. The meal was ingested within 20 minutes after baseline bloods. Bloods were taken at 2, 4, 6, and 8 hours after ingestion of the meal. Subjects were advised to restrict their physical activity to the minimum until the end of the sampling period.

### 2.5. Laboratory Methods

#### 2.5.1. Hormones, Glucose, HbA1c, Lipids, and Apolipoproteins A-I, A-II, and B

Glucose was measured by an enzymatic (hexokinase) method on the Roche P Module (Roche, Stockholm, Sweden) and insulin was measured by electrochemiluminescence immunoassay on the Roche E Module (coefficients of variation (CVs) <5% for both). Glucose and insulin levels were used to calculate HOMA_IR_. Nonesterified fatty acids (NEFA) were measured by a kit using the Randox Colorimetric Method (Randox, Antrim, UK) and analyzed on a Hitachi modular analyzer (Tokyo, Japan) (CV < 5%). Luteinising hormone (LH), follicle stimulating hormone (FSH), sex hormone binding globulin (SHBG), DHEAS, oestradiol, thyroid stimulating hormone (TSH), free thyroxine (fT4), prolactin, and cortisol were measured by standard chemiluminescence immunoassays (CVs < 5% for all). Total testosterone was measured by electrochemiluminescence immunoassay on the Roche E Module. Free androgen index (FAI) was calculated by the following formula: FAI = 100 × total testosterone/SHBG. Androstenedione was measured by radioimmunoassay (CV < 5%). Total-cholesterol, TG, and HDL-C were measured using standard laboratory techniques (CV < 5%). LDL-C was calculated using the Friedewald equation. Apolipoproteins A-I (apoA-I), A-II (apoA-II), and B (apoB) were measured by standard nephelometry on a BNII nephelometer (Dade Behring, Deerfield, IL) (CV < 5%).

#### 2.5.2. ApoB-48 ELISA

ApoB-48 was measured using a commercially available ELISA kit (AKHB48, Gentaur BVBA corporation) and using a modified version of the method described by Lorec et al. [13]. The anti-apoB-48 coated plate is initially washed to remove buffer. The appropriately diluted samples are added to the plate and left to react for one hour. Samples were diluted 1 : 250 to ensure that the concentration was not above the assay range. The plate was subsequently washed 4 times, and biotin-conjugated anti-apoB-48 antibody is added. This binds to human apoB-48 bound to the anti-apoB-48 antibody immobilised on the coated plate. This reaction is stopped after 1 hour and the plate washed 4 times. Finally a peroxidase-conjugated avidin is then added to the plate. The avidin and biotin readily bind to ensure that the horseradish peroxidase enzyme is immobilised on the plate. After washing, the chromogenic substrate tetramethyl benzidine (TMB) is added. This forms a blue colour on reaction with horseradish peroxidase enzyme. The reaction stopper (1 M H_2_SO_4_) is added, resulting in a yellow colour formation which is proportional to substrate concentration. The plate was read at an absorbance of 450 nm. Interassay and intra-assay CVs were 14% and 9%, respectively.

### 2.6. Statistical Analysis

Data are presented as mean ± SEM. Skewed variables were logarithmically transformed to normalise data prior to analysis. Initial comparisons between groups were performed using independent *t*-test. Analysis of covariance (ANCOVA) was used to compare differences between the two groups adjusting for 3 separate covariates: BMI, HOMA_IR_, and *S*_I_. Correlations were made using Pearson's correlation coefficient. Multiple linear regression analysis was used to identify independent contributors to postprandial TG, apoB-48, and HDL-chol. Independent variables that correlated significantly in univariate analysis were entered into the multiple regression models in forward stepwise fashion. Statistical significance was defined as *p* < 0.05.

## 3. Results

### 3.1. Baseline Demographic Data and Hormonal Profile

Table 1 shows the baseline data of both groups of subjects. Women with PCOS had greater BMI, WC, HC, and %BF. All androgens were higher whilst SHBG and FSH were lower in women with PCOS. Women with PCOS were more insulin resistant with a higher HOMA_IR_ (4.40 ± 0.41 *μ*mol^2^/L^2^ in PCOS versus 2.56 ± 0.30 *μ*mol^2^/L^2^ in controls) and lower *S*_I_ (1.99 ± 0.18 × 10^−4^ min^−1^/mU/L in PCOS versus 4.05 ± 0.46 × 10^−4^ min^−1^/mU/L in controls).

### 3.2. Postprandial Lipids, NEFA, and Apolipoprotein

**Figure 1:**
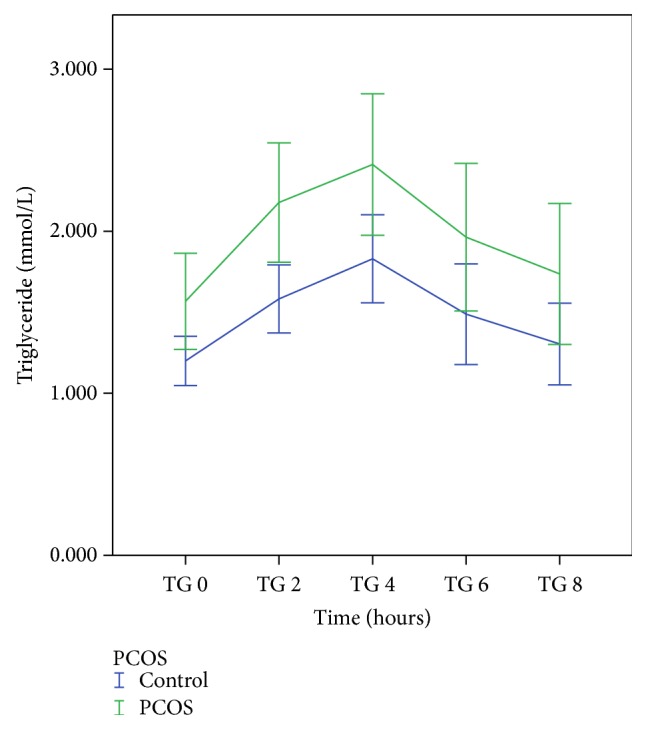
Postprandial triglyceride response in PCOS and control subjects.

**Figure 2:**
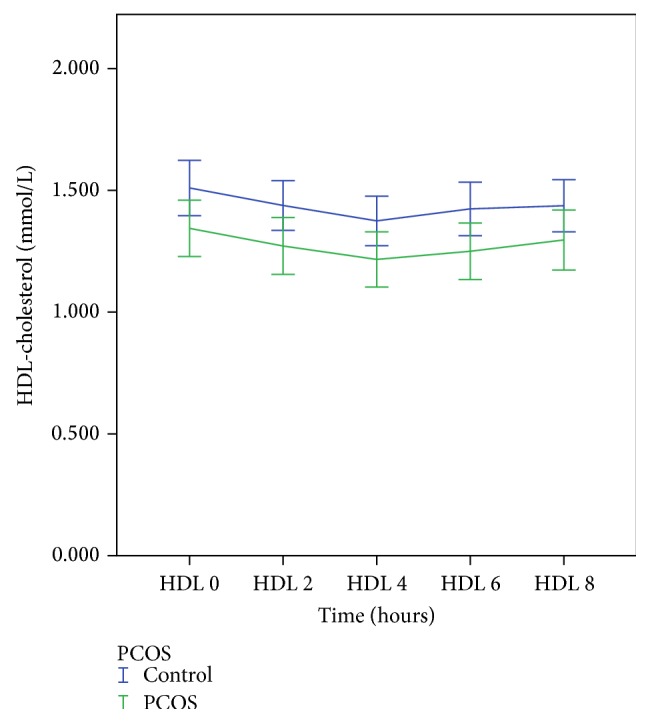
Postprandial HDL-cholesterol response in PCOS and control subjects.

Table 2 shows the pre- and postprandial results of TG, HDL, and NEFA. Postprandial TG at 2 hours was significantly greater in PCOS women (2.18 ± 0.18 mmol/L versus 1.58 ± 0.10 mmol/L, *p* = 0.019) and nonsignificantly greater at the other time points compared to controls (Figure 1). AUC-TG, but not the iAUC-TG, was also significantly higher in women with PCOS. Conversely, HDL-chol was significantly lower in PCOS women compared to controls at *T* = 0, 2, 4, and 6 hours (Figure 2). AUC-HDL was also significantly lower in PCOS compared with controls. NEFA levels did not differ significantly between the two groups. There were no significant differences in apoA-I, apoA-II, apoB, and apoB-48 at any of the time points and the AUC or iAUC of these apolipoproteins between the two groups.

### 3.3. Postprandial Glucose and Insulin

**Figure 3:**
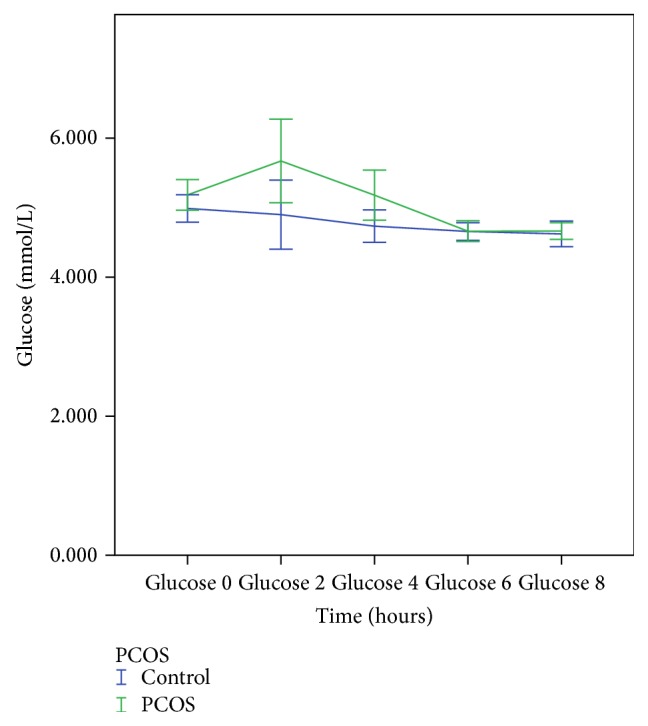
Postprandial glucose response in PCOS and control subjects.

**Figure 4:**
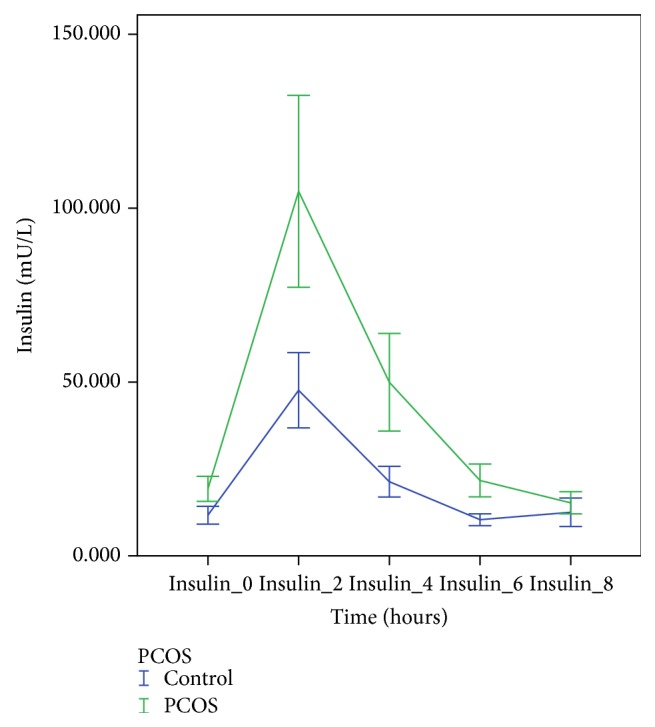
Postprandial insulin response in PCOS and control subjects.

Table 3 shows the pre- and postprandial results for glucose and insulin. Glucose levels were nonsignificantly greater at 2 hours and significantly greater at 4 hours in women with PCOS compared to controls (Figure 3). AUC-glucose was also greater in PCOS. With the exception of insulin at 8 hours, insulin level at all other time points, AUC-insulin, and iAUC-insulin were all greater in women with PCOS (Figure 4).

### 3.4. Comparisons following Adjustment for BMI, HOMA_IR_, and *S*_I_

Tables 4 and 5 show results following adjustment for BMI, HOMA_IR_, and *S*_I_. After adjusting for BMI, androgens remained higher and SHBG lower in PCOS women compared to control subjects (Table 4). There were no differences in TG, HDL, and apoB-48 at any time points nor in AUC-TG, AUC-HDL, AUC-apoB-48. Insulin remained significantly higher at all time points except at 8 hours. AUC-insulin and iAUC-insulin were also significantly higher (Table 5). There were no differences in the other postprandial apolipoproteins.

Following adjustment for HOMA_IR_, androgens and SHBG were significantly different between the two groups (Table 4). ApoB-48 at 4 hours was significantly (*p* = 0.034) higher in the PCOS women (19.8 ± 1.68 *μ*g/mL) compared to controls (15.2 ± 1.64 *μ*g/mL) and insulin at 4 and 6 hours as well as AUC-insulin and iAUC-insulin also remained significantly higher (Table 5).

Finally, following adjustment for *S*_I_, androgens remained significantly greater in PCOS women, but SHBG was no longer significantly different (Table 4). Only insulin at 4 hours was higher in the PCOS women, but AUC-insulin and iAUC-insulin were not different between the two groups (Table 5). ApoB-48 was nonsignificantly (*p* = 0.058) greater at 4 hours in PCOS women (19.2 ± 1.90 *μ*g/mL) compared to controls (16.8 ± 2.00 *μ*g/mL) but not at other time points (Table 5). There were no significant differences in apoB, apoA-I, or apoA-II at any of the time points or in the AUC or iAUC between the two groups.

### 3.5. Correlations and Multiple Regression Analyses

**Figure 5:**
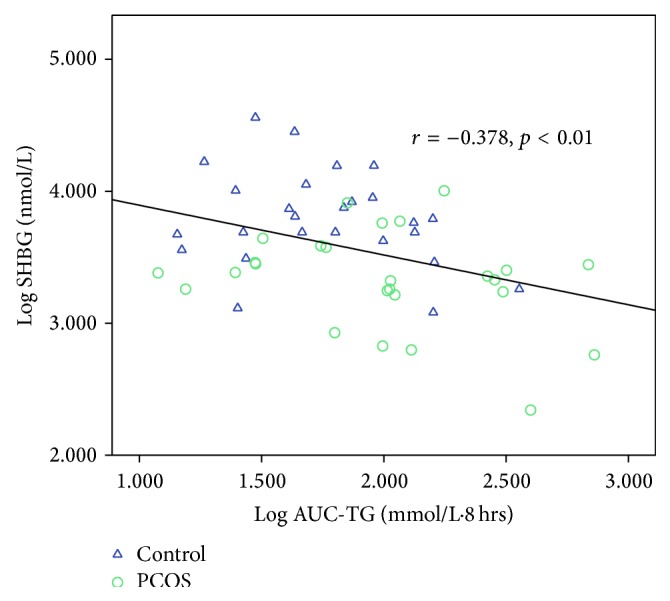
Correlation between AUC-TG and SHBG.

**Figure 6:**
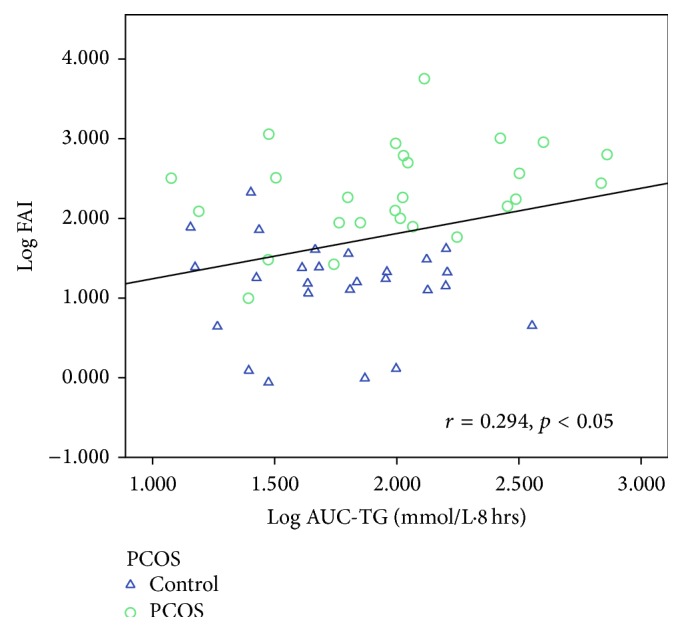
Correlation between AUC-TG and FAI.

**Figure 7:**
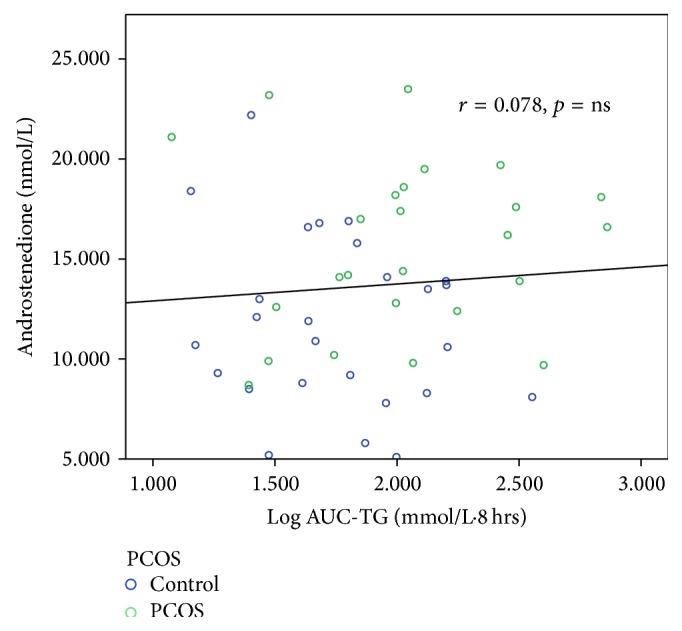
Correlation between AUC-TG and androstenedione.

**Figure 8:**
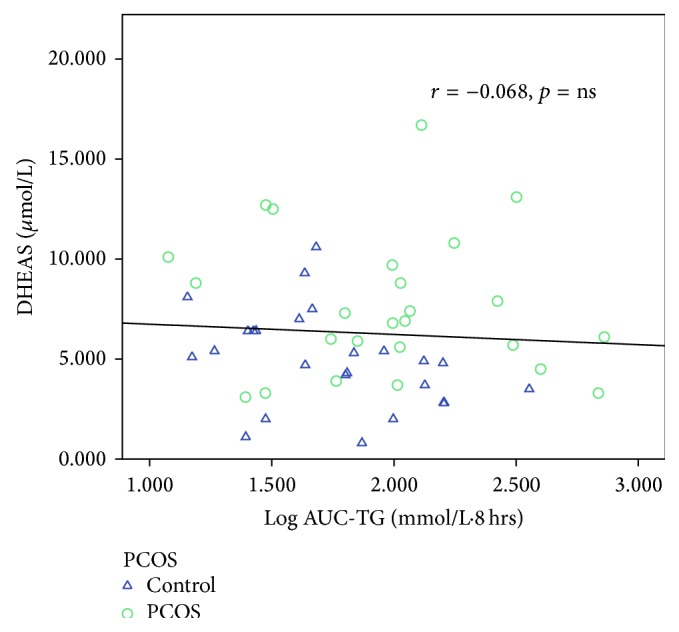
Correlation between AUC-TG and DHEAS.

**Figure 9:**
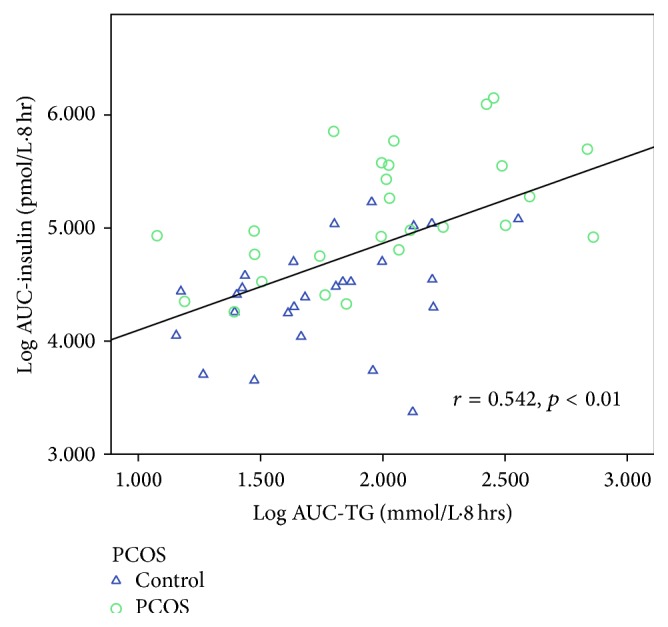
Correlation between AUC-TG and AUC-insulin.

**Figure 10:**
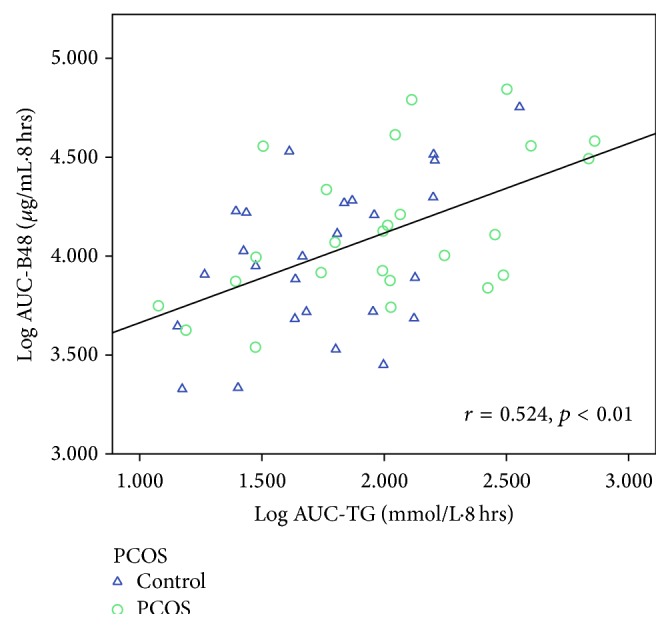
Correlation between AUC-TG and AUC-apoB-48.

Table 6 shows the correlations between AUC-TG, AUC-HDL, AUC-apoB-48, AUC-apoB, AUC-glucose, AUC-insulin, and all other variables. Taking the entire cohort of 52 subjects, AUC-TG correlated positively (*p* < 0.05 for all variables unless otherwise specified) with WC (*r* = 0.314), weight (*r* = 0.357), BMI (*r* = 0.316), and body fat content (*r* = 0.378) and negatively with body water content (*r* = −0.342) and SHBG (*r* = −0.378, *p* < 0.01). AUC-TG did not correlate with androgens except for FAI (*r* = 0.294) and this is likely to be a result of the inverse correlation with SHBG, which causes FAI to be higher. Figures 5–8 show the correlations graphically between AUC-TG and SHBG (Figure 5), FAI (Figure 6), androstenedione (Figure 7), and DHEAS (Figure 8), respectively. As expected, AUC-TG also correlated strongly with HOMA_IR_, *S*_I_ (*r* = −0.601, *p* < 0.01), and AUC-HDL (*r* = −0.540). Insulin at 0, 2, 4, and 6 hours, AUC-insulin, and iAUC-insulin also correlated significantly with AUC-TG (Figure 9). There were also positive correlations between AUC-TG and apoB-48 at 0, 2, 6, and 8 hours, AUC-apoB-48 (Figure 10), apoB at 2, 4, 6, and 8 hours, and AUC-apoB suggesting that both chylomicrons and VLDL contribute to postprandial hypertriglyceridaemia. Glucose at 0 and 2 hours, AUC-glucose, *S*_G_, and DI also correlated with AUC-TG. Stepwise multiple regression analysis revealed that *S*_I_, AUC-apoB-48, and AUC-apoB were independent predictors of AUC-TG, accounting for 55% of the overall variance. This suggests that postprandial chylomicron particles probably play an important role in contributing towards postprandial hypertriglyceridaemia, together with apoB and insulin sensitivity. AUC-TG was the only independent predictor of AUC-apoB-48, explaining 25.8% of the variance.

AUC-HDL correlated significantly negatively with WC (*r* = −0.483, *p* < 0.01), HC (*r* = −0.285), weight (*r* = −0.439, *p* < 0.01), BMI (*r* = −0.456, *p* < 0.01), body fat (*r* = −0.444), FAI (*r* = −0.350), HOMA_IR_ (*r* = −0.499, *p* < 0.01), AUC-TG (*r* = −0.540, *p* < 0.01), AUC-glucose (*r* = −0.309), and AUC-insulin (*r* = −0.488, *p* < 0.01). AUC-HDL correlated significantly positively with body water (*r* = 0.389), SHBG (*r* = 0.443, *p* < 0.01), and *S*_I_ (*r* = 0.454). AUC-HDL also correlated negatively with TG at all time points, iAUC-TG, iAUC-B48, apoA-I at 2, 4, and 6 hours and AUC-apoA-I, glucose at 0 and 2 hours, AUC-glucose, insulin at 0, 2, 4, and 6 hours, AUC, and iAUC-insulin (results not shown). There was a nonsignificant negative correlation with androstenedione (*r* = −0.27, *p* = 0.055). AUC-TG and WC were the only independent predictors of AUC-HDL, explaining 37.2% of the variance using multiple regression analysis.

As expected, significant correlations were found between AUC-insulin and SHBG. AUC-insulin also correlated strongly with FAI, AUC-TG, AUC-HDL, AUC-glucose, PCOS status, and anthropometrics. Finally AUC-insulin was negatively correlated with DI, suggesting a correlation with possible subsequent *β*-cell function failure and development of diabetes.

## 4. Discussion

Dyslipidaemia, including both increased TG and low HDL-cholesterol, is common in PCOS [16, 17] and is a well-recognized feature of the metabolic syndrome which confers increased risk of cardiovascular disease [18, 19]. Hypertriglyceridaemia signifies the presence of excess TRL. TRL consists of hepatically derived VLDL, characterised by the presence of apoB-100, and intestinally derived chylomicrons, which contain apoB-48. Both VLDL and chylomicrons share a common lipolytic pathway and are hydrolysed by lipoprotein lipase (LPL) and there are a number of described receptor mediated pathways through which these particles may eventually be cleared from the circulation [12]. It is postulated that an excess production and/or a delay in clearance of postprandial lipoproteins of both TRL particles may be implicated in atherosclerosis [20, 21].

To the best of the author's knowledge, this is the first study to compare the postprandial responses in obese PCOS women with that of obese control subjects. Although some postprandial studies in PCOS women have been done previously, this is the first one to evaluate the contribution of the two main metabolic/endocrine disturbances in PCOS women—IR and hyperandrogenaemia—to postprandial dyslipidaemia in obese women with PCOS. Both are implicated in accelerated atherosclerosis but the relative contribution of either is a subject of controversy [22–24]. Obesity, in particular central obesity, is strongly associated with insulin resistance, whilst androgens are also associated with an android distribution of fat. In a previous study by Velázquez M et al. a strong correlation between postprandial triglycerides and waist-to-hip ratio was demonstrated. That study evaluated overweight (BMI 27.41 ± 0.50 kg/m^2^) PCOS women and compared them to lean PCOS women and lean controls. Only free testosterone was measured, and there was no analysis between this and postprandial hypertriglyceridaemia [10]. A separate study by Bahceci et al. evaluated postprandial responses to an oral fat tolerance test, in lean PCOS women with lean controls (BMI 23.5 ± 2.6 kg/m^2^ versus 23.1 ± 4.0 kg/m^2^; *p* > 0.05). They showed that PCOS women had higher baseline insulin, HOMA_IR_, AUC-TG, AUC-total-cholesterol, AUC-VLDL-cholesterol, and AUC-apoB. Surprisingly, though, AUC-insulin did not differ between the two groups. Again, no analyses were reported between androgens and postprandial lipids [11].

In this study, comparing obese women with PCOS with obese control subjects, AUC-TG, AUC-HDL, AUC-glucose, and AUC-insulin were all significantly different. HDL-chol and insulin were different at most of the time points, but TG was only different at 2 hours. Independent predictors of AUC-TG included *S*_I_, AUC-apoB-48, and AUC-apoB, accounting for 55% of the variance. This suggests that postprandial chylomicron particles have an important role to play in postprandial hypertriglyceridaemia, together with insulin sensitivity and apoB. On the other hand, AUC-TG was the only independent predictor of AUC-apoB-48, explaining 25.8% of the variance.

Following adjustment for BMI, lipid differences were no longer noted but insulin levels at all time points and AUC-insulin and iAUC-insulin were still much greater in PCOS women consistent with the fact that PCOS women are more insulin resistant than their BMI matched counterparts [25, 26]. In this study, IR was determined by the more convenient HOMA_IR_ and insulin sensitivity was determined by the more sophisticated fsIVGTT derived *S*_I_. When comparisons were made adjusting for HOMA_IR_, postprandial insulins were still significantly higher in the PCOS group. However, these differences were largely no longer present when adjustments were made for *S*_I_, which may be a better marker for insulin sensitivity than HOMA_IR_, since the latter is calculated from fasting values of insulin and glucose.

There was only weak or no correlation between androgens and TG, HDL-chol, apoB-48, apoA-I, apoA-II, and apoB. There were very strong correlations between SHBG and TG, HDL-chol, AUC-apoB-48, AUC-apoB, and AUC-apoA-II. FAI but not testosterone* per se* also strongly correlated with HDL at all time points, TG at 0 and 2 hours, and both AUC-HDL-chol and AUC-TG. This suggests that IR, and perhaps obesity, both of which are strongly negatively correlated with SHBG, rather than androgens, may be the major determinant of postprandial lipids.

This study had certain limitations. PCOS women and the controls were not entirely matched for BMI. However, both groups were obese and results were analyzed with BMI as a covariate. The two groups did not match for IR as assessed by HOMA_IR_ nor *S*_I_ as assessed by the fsIVGTT. However this is to be expected based on the well accepted notion that women with PCOS are more insulin resistant than BMI matched counterparts. In addition, after using HOMA_IR_ and *S*_I_ as covariates, results still revealed that hyperinsulinaemia and possibly a low SHBG level are the main factors influencing postprandial hypertriglyceridaemia. Postprandial chylomicrons also appear to play a significant role in postprandial hypertriglyceridaemia in premenopausal women with and without PCOS.

In conclusion, this study provides evidence that, in obese women with PCOS, IR plays a more important role than hyperandrogenaemia in postprandial dyslipidaemia and cardiovascular risk. Targeting obesity and thereby improving IR by lifestyle measures and perhaps the use of agents such as metformin should be a priority in the treatment of obese PCOS women.

## Figures and Tables

**Table 1:** Baseline characteristics of all subjects (*n* = 52).

	PCOS (*n* = 26)	Controls (*n* = 26)	*p*
Age (yrs)	30.4 ± 1.2	34.1 ± 1.5	0.063
Systolic BP (mmHg)	126.3 ± 1.8	121.6 ± 2.5	0.131
Diastolic BP (mmHg)	79.0 ± 1.1	77.8 ± 2.3	0.652
Waist (cm)	112.0 ± 2.2	99.8 ± 2.4	<0.001
Hip (cm)	120.5 ± 1.7	110.9 ± 1.9	<0.001
Waist : Hip	0.930 ± 0.014	0.900 ± 0.014	0.139
Weight (kg)	95.4 ± 2.6	83.2 ± 2.8	0.003
BMI (kg/m^2^)	36.8 ± 0.9	31.5 ± 1.0	<0.001
Smoking (%)	26	31	0.696
% Body fat mass (%)	40.4 ± 1.3	33.6 ± 2.0	0.006
T (nmol/L)^*∗*^	3.22 ± 0.30	1.57 ± 0.13	<0.001
SHBG (nmol/L)^*∗*^	29.8 ± 2.00	47.4 ± 3.48	<0.001
FAI^*∗*^	12.2 ± 1.59	3.67 ± 0.39	<0.001
Androstenedione (nmol/L)	15.58 ± 0.84	11.82 ± 0.84	0.003
DHEAS (*μ*mol/L)	7.62 ± 0.71	4.98 ± 0.49	0.003
FSH (IU/L)^*∗*^	5.54 ± 0.35	8.93 ± 0.90	<0.001
LH (IU/L)^*∗*^	6.37 ± 0.74	7.49 ± 1.41	0.654
HOMA_IR_ (*μ*mol^2^/L^2^)^*∗*^	4.40 ± 0.41	2.56 ± 0.30	<0.001
*S*_I_ (×10^−4^ min^−1^/mU/L)^*∗*^	1.99 ± 0.18	4.05 ± 0.46	<0.001
*S*_G_ (min^−1^)	0.02 ± 0.00	0.02 ± 0.00	0.352
AIR_G_ (mU·L^−1^·min)^*∗*^	985 ± 171	613 ± 85	0.207
DI	1756 ± 281	2308 ± 358	0.226

^*∗*^ *t*-tests analysis after log transformation.

**Table 2:** Postprandial triglycerides, HDL-chol, NEFA, and apolipoproteins.

	PCOS (*n* = 26)	Controls (*n* = 26)	*p*
TG 0 hrs (mmol/L)^*∗*^	1.57 ± 0.15	1.20 ± 0.08	0.075
TG 2 hrs (mmol/L)^*∗*^	2.18 ± 0.18	1.58 ± 0.10	0.019
TG 4 hrs (mmol/L)^*∗*^	2.41 ± 0.22	1.83 ± 0.14	0.057
TG 6 hrs (mmol/L)^*∗*^	1.96 ± 0.23	1.49 ± 0.16	0.074
TG 8 hrs (mmol/L)^*∗*^	1.74 ± 0.22	1.30 ± 0.13	0.129
AUC-TG (mmol/L·8 hrs)^*∗*^	8.20 ± 0.77	6.15 ± 0.45	0.041
iAUC-TG (mmol/L·8 hrs)	1.93 ± 0.28	1.35 ± 0.24	0.127
HDL-chol 0 hrs (mmol/L)	1.34 ± 0.06	1.51 ± 0.06	0.046
HDL-chol 2 hrs (mmol/L)	1.27 ± 0.06	1.44 ± 0.05	0.037
HDL-chol 4 hrs (mmol/L)	1.22 ± 0.06	1.37 ± 0.05	0.043
HDL-chol 6 hrs (mmol/L)	1.25 ± 0.06	1.42 ± 0.05	0.034
HDL-chol 8 hrs (mmol/L)	1.30 ± 0.06	1.44 ± 0.05	0.092
AUC-HDL-chol (mmol/L·8 hrs)	5.06 ± 0.23	5.71 ± 0.21	0.041
iAUC-HDL-chol (mmol/L·8 hrs)	−0.32 ± 0.03	−0.33 ± 0.07	0.883
NEFA 0 hrs (mmol/L)	0.65 ± 0.04	0.61 ± 0.05	0.644
NEFA 2 hrs (mmol/L)	0.19 ± 0.02	0.18 ± 0.05	0.658
NEFA 4 hrs (mmol/L)	0.33 ± 0.03	0.36 ± 0.05	0.524
NEFA 6 hrs (mmol/L)	0.62 ± 0.05	0.67 ± 0.05	0.544
NEFA 8 hrs (mmol/L)	0.81 ± 0.05	0.84 ± 0.05	0.698
AUC NEFA (mmol/L·8 hrs)	1.87 ± 0.10	1.94 ± 0.05	0.681
iAUC NEFA (mmol/L·8 hrs)	−0.72 ± 0.14	−0.52 ± 0.05	0.400
ApoB-48 0 hrs (*μ*g/mL)^*∗*^	11.9 ± 1.45	9.89 ± 1.29	0.252
ApoB-48 2 hrs (*μ*g/mL)^*∗*^	17.0 ± 1.43	15.7 ± 1.08	0.468
ApoB-48 4 hrs (*μ*g/mL)^*∗*^	19.2 ± 1.56	15.8 ± 1.55	0.058
ApoB-48 6 hrs (*μ*g/mL)^*∗*^	18.3 ± 1.70	15.8 ± 1.93	0.184
ApoB-48 8 hrs (*μ*g/mL)^*∗*^	12.9 ± 1.34	11.3 ± 1.48	0.239
AUC-apoB-48 (*μ*g/mL·8 hrs)^*∗*^	66.4 ± 5.32	57.6 ± 4.41	0.157
iAUC-apoB-48 (*μ*g/mL·8 hrs)	19.35 ± 3.38	18.27 ± 2.45	0.796
ApoB 0 hrs (g/dL)	0.73 ± 0.07	0.82 ± 0.06	0.328
ApoB 2 hrs (g/dL)	0.76 ± 0.07	0.73 ± 0.06	0.816
ApoB 4 hrs (g/dL)	0.83 ± 0.06	0.76 ± 0.06	0.364
ApoB 6 hrs (g/dL)	0.79 ± 0.05	0.77 ± 0.05	0.779
ApoB 8 hrs (g/dL)	0.83 ± 0.05	0.79 ± 0.05	0.603
AUC-apoB (g/dL·8 hrs)	3.16 ± 0.17	3.07 ± 0.14	0.688
iAUC-apoB (g/dL·8 hrs)	0.22 ± 0.22	−0.22 ± 0.19	0.143
ApoA-I 0 hrs (g/dL)	1.15 ± 0.07	1.10 ± 0.10	0.679
ApoA-I 2 hrs (g/dL)	1.04 ± 0.09	1.14 ± 0.09	0.437
ApoA-I 4 hrs (g/dL)	1.19 ± 0.08	1.28 ± 0.08	0.465
ApoA-I 6 hrs (g/dL)	1.18 ± 0.08	1.22 ± 0.08	0.715
ApoA-I 8 hrs (g/dL)	1.08 ± 0.08	1.16 ± 0.09	0.481
AUC-apoA-I (g/dL·8 hrs)	4.52 ± 0.19	4.75 ± 0.18	0.391
iAUC-apoA-I (g/dL·8 hrs)	−0.08 ± 0.30	0.35 ± 0.34	0.342
ApoA-II 0 hrs (g/dL)	0.32 ± 0.02	0.30 ± 0.03	0.544
ApoA-II 2 hrs (g/dL)	0.29 ± 0.03	0.30 ± 0.02	0.796
ApoA-II 4 hrs (g/dL)	0.26 ± 0.03	0.25 ± 0.03	0.764
ApoA-II 6 hrs (g/dL)	0.26 ± 0.02	0.30 ± 0.02	0.195
ApoA-II 8 hrs (g/dL)	0.25 ± 0.02	0.26 ± 0.02	0.761
AUC-apoA-II (g/dL·8 hrs)	1.10 ± 0.04	1.13 ± 0.04	0.657
iAUC-apoA-II (g/dL·8 hrs)	−0.19 ± 0.09	−0.08 ± 0.09	0.410

^*∗*^ *t*-tests analysis after log transformation.

**Table 3:** Postprandial glucose and insulin.

	PCOS (*n* = 26)	Controls (*n* = 26)	*p*
Glucose 0 hrs (mmol/L)	5.18 ± 0.11	4.99 ± 0.10	0.191
Glucose 2 hrs (mmol/L)	5.67 ± 0.30	4.90 ± 0.25	0.053
Glucose 4 hrs (mmol/L)	5.18 ± 0.18	4.73 ± 0.12	0.044
Glucose 6 hrs (mmol/L)	4.66 ± 0.08	4.66 ± 0.06	0.959
Glucose 8 hrs (mmol/L)	4.66 ± 0.06	4.62 ± 0.09	0.713
AUC-glucose (mmol/L·8 hrs)	20.50 ± 0.48	19.10 ± 0.38	0.024
iAUC-glucose (mmol/L·8 hrs)	−0.33 ± 0.32	−0.86 ± 0.40	0.306
Insulin 0 hrs (mU/L)^*∗*^	19.3 ± 1.80	11.7 ± 1.28	<0.001
Insulin 2 hrs (mU/L)^*∗*^	105 ± 13.8	47.6 ± 5.41	<0.001
Insulin 4 hrs (mU/L)^*∗*^	49.9 ± 7.02	21.4 ± 2.21	<0.001
Insulin 6 hrs (mU/L)^*∗*^	21.7 ± 2.35	10.4 ± 0.85	<0.001
Insulin 8 hrs (mU/L)^*∗*^	15.3 ± 1.59	12.6 ± 2.05	0.084
AUC-insulin (mU/L·8 hrs)^*∗*^	194 ± 21.6	91.5 ± 8.03	<0.001
iAUC-insulin (mU/L·8 hrs)^*∗*^	117 ± 17.5	44.8 ± 5.70	<0.001

^*∗*^ *t*-tests analysis after log transformation.

**Table 4:** Baseline characteristics adjusted for BMI, HOMA_IR_, and log *S*_I_.

	Covariate: BMI		Covariate: HOMA_IR_		Covariate: *S*_I_	
	PCOS	Controls	PCOS	Controls	PCOS	Controls
Systolic BP (mmHg)	124.6 ± 2.2	123.3 ± 2.2	124.4 ± 2.2	123.5 ± 2.2	125.0 ± 2.4	123.0 ± 2.6
Diastolic BP (mmHg)	78.0 ± 1.9	78.9 ± 1.9	78.6 ± 2.0	78.2 ± 2.0	78.6 ± 2.1	78.9 ± 2.3
Waist (cm)	107.3 ± 1.5	104.5 ± 1.5	109.0 ± 2.2	102.8 ± 2.2	108.8 ± 2.4	104.3 ± 2.6
Hip (cm)	116.6 ± 1.1	114.8 ± 1.1	118.7 ± 1.9^a^	112.6 ± 1.9	118.4 ± 2.0	113.3 ± 2.2
Waist : Hip	0.920 ± 0.015	0.910 ± 0.015	0.917 ± 0.015	0.913 ± 0.015	0.919 ± 0.016	0.921 ± 0.017
Weight (kg)	89.2 ± 1.4	89.5 ± 1.4	91.2 ± 2.5	87.4 ± 2.5	91.0 ± 2.8	88.9 ± 3.0
BMI (kg/m^2^)	—		35.3 ± 1.0	32.9 ± 0.9	35.4 ± 1.1	33.7 ± 1.2
% Body fat mass (%)	37.6 ± 1.2	36.2 ± 1.2	38.2 ± 1.7	35.7 ± 1.7	38.5 ± 1.8	36.2 ± 1.9
T (nmol/L)^*∗*^	3.37 ± 0.25^b^	1.52 ± 0.24	3.24 ± 0.25^b^	1.56 ± 0.25	3.49 ± 0.27^b^	1.30 ± 0.279
SHBG (nmol/L)^*∗*^	32.2 ± 3.04^b^	45.2 ± 2.98	31.4 ± 2.98^b^	45.7 ± 2.98	34.2 ± 3.19	43.4 ± 3.45
FAI^*∗*^	12.2 ± 1.25^b^	4.08 ± 1.22	12.2 ± 1.24^b^	3.70 ± 1.24	12.5 ± 1.38^b^	3.30 ± 1.50
Androstenedione (nmol/L)	15.67 ± 0.9^b^	11.99 ± 0.89	15.07 ± 0.92^a^	12.30 ± 0.90	15.98 ± 0.99^b^	11.37 ± 1.05
DHEAS (*μ*mol/L)	8.00 ± 0.68^b^	4.80 ± 0.66	7.88 ± 0.66	4.72 ± 0.66	8.55 ± 0.66^b^	3.80 ± 0.72
HOMA_IR_ (*μ*mol^2^/L^2^)^*∗*^	3.99 ± 0.35^a^	3.02 ± 0.34	—		3.91 ± 0.38	3.39 ± 0.41
*S*_I_ (×10^−4^ min^−1^/mU/L)^*∗*^	2.23 ± 0.31^b^	3.78 ± 0.34	2.33 ± 0.30^a^	3.66 ± 0.32	—	
*S*_G_ (min^−1^)	0.02 ± 0.00	0.02 ± 0.00	0.02 ± 0.00	0.02 ± 0.00	0.02 ± 0.00	0.02 ± 0.00
AIR_G_ (mU·L^−1^·min)^*∗*^	986 ± 144	612 ± 155	867 ± 133	747 ± 143	909 ± 149	700 ± 161
DI	1846 ± 321	2206 ± 345	1893 ± 326	2151 ± 351	2174 ± 312	1831 ± 338

^*∗*^ANCOVA analysis after log transformation. ^a^ *p* < 0.05 versus controls. ^b^ *p* < 0.01 versus controls.

**Table 5:** Postprandial TG, HDL, apoB-48, glucose, and insulin levels adjusted for BMI, HOMA_IR_, and *S*_I_.

	Covariate: BMI		Covariate: HOMA_IR_		Covariate: *S*_I_	
	PCOS	Controls	PCOS	Controls	PCOS	Controls
TG 0 hrs (mmol/L)^*∗*^	1.54 ± 0.13	1.25 ± 0.12	1.40 ± 0.11	1.36 ± 0.11	1.47 ± 0.13	1.33 ± 0.14
TG 2 hrs (mmol/L)^*∗*^	2.14 ± 0.16	1.66 ± 0.16	1.97 ± 0.14	1.79 ± 0.14	2.04 ± 0.17	1.73 ± 0.18
TG 4 hrs (mmol/L)^*∗*^	2.37 ± 0.20	1.90 ± 0.19	2.14 ± 0.16	2.10 ± 0.16	2.24 ± 0.20	2.10 ± 0.22
TG 6 hrs (mmol/L)^*∗*^	1.92 ± 0.21	1.57 ± 0.21	1.70 ± 0.18	1.75 ± 0.18	1.80 ± 0.22	1.77 ± 0.24
TG 8 hrs (mmol/L)^*∗*^	1.71 ± 0.19	1.37 ± 0.19	1.48 ± 0.16	1.56 ± 0.16	1.60 ± 0.20	1.56 ± 0.22
AUC-TG (mmol/L·8 hrs)^*∗*^	8.06 ± 0.68	6.45 ± 0.66	7.25 ± 0.54	7.10 ± 0.54	7.61 ± 0.70	7.04 ± 0.76
iAUC-TG (mmol/L·8 hrs)	1.87 ± 0.29	1.45 ± 0.29	1.63 ± 0.27	1.65 ± 0.27	1.64 ± 0.31	1.82 ± 0.33
HDL-chol 0 hrs (mmol/L)	1.40 ± 0.06	1.45 ± 0.06	1.41 ± 0.06	1.44 ± 0.06	1.40 ± 0.06	1.38 ± 0.07
HDL-chol 2 hrs (mmol/L)	1.33 ± 0.06	1.38 ± 0.06	1.34 ± 0.05	1.37 ± 0.05	1.34 ± 0.06	1.33 ± 0.07
HDL-chol 4 hrs (mmol/L)	1.27 ± 0.06	1.32 ± 0.05	1.28 ± 0.05	1.31 ± 0.05	1.28 ± 0.06	1.27 ± 0.06
HDL-chol 6 hrs (mmol/L)	1.30 ± 0.06	1.37 ± 0.06	1.32 ± 0.06	1.36 ± 0.06	1.31 ± 0.06	1.31 ± 0.07
HDL-chol 8 hrs (mmol/L)	1.35 ± 0.06	1.38 ± 0.06	1.36 ± 0.06	1.37 ± 0.06	1.36 ± 0.06	1.32 ± 0.07
AUC-HDL-chol (mmol/L·8 hrs)	5.28 ± 0.23	5.49 ± 0.22	5.33 ± 0.22	5.44 ± 0.22	5.31 ± 0.24	5.26 ± 0.26
iAUC-HDL-chol (mmol/L·8 hrs)	−0.33 ± 0.06	−0.31 ± 0.06	−0.33 ± 0.06	−0.32 ± 0.06	−0.30 ± 0.06	−0.27 ± 0.07
ApoB-48 0 hrs (*μ*g/mL)^*∗*^	12.4 ± 1.54	9.70 ± 1.47	11.9 ± 1.49	9.86 ± 1.46	11.9 ± 1.70	10.4 ± 1.80
ApoB-48 2 hrs (*μ*g/mL)^*∗*^	17.3 ± 1.43	15.6 ± 1.36	17.0 ± 1.37	15.7 ± 1.34	16.5 ± 1.53	16.9 ± 1.62
ApoB-48 4 hrs (*μ*g/mL)^*∗*^	19.6 ± 1.77	15.5 ± 1.69	19.8 ± 1.68^a^	15.2 ± 1.64	19.2 ± 1.90	16.8 ± 2.00
ApoB-48 6 hrs (*μ*g/mL)^*∗*^	18.9 ± 2.06	15.6 ± 1.97	17.2 ± 1.94	16.9 ± 1.90	17.9 ± 2.22	17.5 ± 2.34
ApoB-48 8 hrs (*μ*g/mL)^*∗*^	13.2 ± 1.60	11.2 ± 1.53	11.7 ± 1.47	12.4 ± 1.44	12.3 ± 1.70	12.5 ± 1.79
Log ApoB-48 (*μ*g/mL·8 hrs)^*∗*^	67.8 ± 5.51	57.1 ± 5.26	65.6 ± 5.30	58.4 ± 5.18	65.0 ± 5.97	62.5 ± 6.30
iAUC apoB-48 (*μ*g/mL·8 hrs)	19.02 ± 3.33	18.31 ± 3.18	17.57 ± 3.19	19.98 ± 3.12	17.92 ± 3.70	21.06 ± 3.91
Insulin 0 hrs (pmol/L)^*∗*^	17.9 ± 1.56^a^	13.4 ± 1.2	15.4 ± 0.30	15.6 ± 0.30	17.5 ± 1.66	15.1 ± 1.79
Insulin 2 hrs (pmol/L)^*∗*^	95.7 ± 10.3^a^	59.5 ± 10.1	88.7 ± 8.91	63.8 ± 8.91	91.6 ± 11.3	64.1 ± 12.2
Insulin 4 hrs (pmol/L)^*∗*^	47.6 ± 5.59^b^	24.6 ± 5.47	43.7 ± 4.92^a^	27.6 ± 4.92	45.7 ± 5.94^b^	27.7 ± 6.42
Insulin 6 hrs (pmol/L)^*∗*^	20.9 ± 1.90^b^	11.6 ± 1.86	19.2 ± 1.57^b^	12.9 ± 1.57	19.9 ± 1.94	13.2 ± 2.11
Insulin 8 hrs (pmol/L)^*∗*^	13.6 ± 1.89	14.4 ± 1.84	13.4 ± 1.81	14.3 ± 1.81	14.1 ± 2.17	14.7 ± 2.35
AUC-insulin (pmol/L·8 hrs)^*∗*^	180 ± 16.2^b^	110 ± 15.8	166 ± 13.0^a^	119 ± 13.0	173 ± 17.5	120 ± 18.9
iAUC-insulin (pmol/L·8 hrs)^*∗*^	108 ± 13.5^a^	55.9 ± 13.2	104 ± 13.0^a^	57.0 ± 13.0	103 ± 14.7	59.6 ± 15.9

^*∗*^ANCOVA analysis after log transformation. ^a^ *p* < 0.05 versus controls. ^b^ *p* < 0.01 versus controls.

**Table 6:** Correlations between AUC-TG, AUC-HDL-chol, AUC-apoB-48, AUC-apoB, AUC-glucose, AUC-insulin, and other variables.

	AUC-TG^*∗*^ (mmol/L·8 hrs)	AUC-HDL (mmol/L·8 hrs)	AUC-apoB-48^*∗*^ (*μ*g/mL·8 hrs)	AUC-B (g/dL·8 hrs)	AUC-glucose (mmol/L·8 hrs)	AUC-insulin^*∗*^ (pmol/L·8 hrs)
AUC-TG^*∗*^ (mmol/L·8 hrs)	1.000	−0.540^b^	0.524^b^	0.484^b^	0.304^a^	0.542^b^
AUC-HDL-chol (mmol/L·8 hrs)	−0.540^b^	1.000	−0.229	−0.225	−0.309^a^	−0.488^b^
AUC-apoB-48^*∗*^ (*μ*g/mL·8 hrs)	0.524^b^	−0.229	1.000	0.298^a^	0.171	0.165
AUC-apoB (g/dL·8 hrs)	0.484^b^	−0.225	0.298^a^	1.000	0.038	0.206
AUC-glucose (mmol/L·8 hrs)	0.304^a^	−0.309^a^	0.171	0.038	1.000	0.634^b^
AUC-insulin^*∗*^ (pmol/L·8 hrs)	0.542^b^	−0.488^b^	0.165	0.206	0.634^b^	1.000

Age, PCOS status						
Age (years)	0.125	−0.010	−0.025	0.134	0.342^a^	−0.014
PCOS	0.284^a^	−0.284^a^	0.201	0.057	0.315^a^	0.579^b^

Androgens, SHBG						
T^*∗*^ (nmol/L)	0.133	−0.163	0.115	−0.023	0.092	0.331
SHBG^*∗*^ (nmol/L)	−0.378^b^	0.443^b^	−0.277^a^	−0.277^a^	−0.305^a^	−0.550^b^
FAI^*∗*^	0.294^a^	−0.350^a^	0.227	0.125	0.226	0.530^b^
Androstenedione (nmol/L)	0.078	−0.271	−0.014	−0.056	0.146	0.416
DHEAS (*μ*mol/L)	−0.068	−0.048	0.108	−0.289^a^	0.125	0.158

Anthropometrics						
Waist (cm)	0.314^a^	−0.483^b^	0.063	0.080	0.372^b^	0.661^b^
Hip (cm)	0.216	−0.285^a^	−0.064	0.154	0.356^a^	0.609^b^
Waist : Hip	0.237	−0.439^b^	0.163	−0.047	0.193	0.360^a^
Weight (kg)	0.357^a^	−0.378^b^	0.059	0.276	0.271	0.639^b^
BMI (kg/m^2^)	0.316^a^	−0.456^b^	0.033	0.184	0.414^b^	0.686^b^
% Body fat mass (%)	0.378^a^	−0.444^b^	0.009	0.164	0.368^a^	0.539^b^
% Body lean mass (%)	−0.128	0.240	0.085	−0.152	−0.348^a^	−0.381^a^
% Body water (%)	−0.342^a^	0.389^a^	−0.031	−0.178	−0.314	−0.487^b^

IR, insulin sensitivity, and variables derived from the fsIVGTT						
HOMA_IR_^*∗*^ (*μ*mol^2^/L^2^)	0.568^b^	−0.499^b^	0.130	0.229	0.366^b^	0.800^b^
*S*_I_ (×10^−4^ min^−1^/mU/L)^*∗*^	−0.601^b^	0.454^b^	−0.223	−0.262	−0.453^b^	−0.810^b^
*S*_G_ (min^−1^)	−0.364^a^	0.159	−0.266	−0.232	−0.083	−0.171
AIR_G_ (mU·L^−1^·min)^*∗*^	0.029	−0.090	−0.282	0.100	−0.410^b^	0.153
DI	−0.364^a^	0.226	−0.328^a^	−0.139	−0.558^b^	−0.391^b^

^*∗*^Correlations after log transformation.

^a^ *p* < 0.05. ^b^ *p* < 0.01.
